# Leveraging Academic Health Departments for Place-Based Disaster Response: Lessons from Helene in Western North Carolina

**DOI:** 10.13023/jah.0703.12

**Published:** 2025-09-01

**Authors:** Danny Scalise, Sarah B. Thach, Ellis V. Matheson

**Affiliations:** Burke County Health Department; University of North Carolina at Chapel Hill; Buncombe County Department of Health and Human Services

**Keywords:** Academic partnerships, Appalachia, disaster recovery, public health departments

## Abstract

This article examines the role of two Academic Health Departments (AHDs) in Buncombe and Burke Counties, North Carolina in responding to Hurricane Helene in 2024. The partnership between local health departments and the UNC Asheville – UNC Gillings Master of Public Health (MPH) Program fostered a place-based approach to public health. After the hurricane disrupted regional infrastructure, the AHDs integrated students and faculty into recovery efforts. This paper identifies key lessons for strengthening public health preparedness through academic-practice partnerships and highlights the importance of embedding education in local contexts.

## INTRODUCTION

Academic-practice partnerships (i.e., collaborations between local or state health departments and university public health training programs) are a well-established model for enhancing public health systems, including disaster response,^1^,^2^ through shared teaching of students, service, and research. Academic Health Departments (AHDs) formalize these collaborations between public health agencies and academic institutions.^3^ In Western North Carolina (WNC), Buncombe County Health Department and Burke County Public Health are developing AHDs with the UNC Asheville – UNC Gillings Master of Public Health (MPH) Program, among other institutions. Within this partnership, the Buncombe health director teaches two courses in the MPH program, and students visit the Burke County health department. Both individuals and teams of students engage with both health departments on community projects. This article documents the development of these partnerships and their role in health departments’ emergency response to Hurricane Helene.^4^–^6^

### Literature Review

The concept of AHDs has been widely studied in the context of workforce development, translational research (translating basic research into applications that help people), and service-learning. Studies highlight that AHDs improve public health training, foster innovation, and support evidence-based practice.^7^ A place-based public health approach emphasizes community-led interventions, informed by local context, that simultaneously enhance individual health, community capacity, and just systems.^8^ Disaster preparedness literature stresses the value of pre-existing partnerships for coordinated response.^9^–^11^ However, limited research exists on how AHDs contribute to disaster response, particularly in rural or underserved areas.

## METHODS

This case study examines the role of AHDs in recovering from Hurricane Helene. The two health directors and director of the UNC Asheville – UNC Gillings MPH program met together in April 2025 to reflect on Helene and brainstorm lessons learned for AHDs related to preparedness and response activities.

## RESULTS

The research team identified the following themes.

### Benefits of Preexisting Relationships

Preexisting relationships between academic institutions and health departments facilitated university faculty and students’ prompt and meaningful engagement in health departments’ recovery efforts. In the chaos that follows a disaster, there is little time or bandwidth to forge new relationships. Quite simply, people reach out to those whose contact information is already known and easily accessible, particularly those recorded in their cell phones.

### Student Engagement and Involvement

The educational disruption prompted innovations in course delivery that allowed for more student engagement in community relief and recovery activities. For example: while engaging in recovery efforts, students learned about needs assessment, public communication, and logistical support in real time. One team of students helped design a lead education campaign. Another developed an Emergency Services plan to improve emergency communication through dissemination of hand crank radios.

### Customized University Support

The place-based model allowed for tailored interventions rooted in local knowledge. Health departments most appreciated academic support that involved listening to local needs, providing relevant expertise, and facilitating communication with other communities who had experienced similar disasters.

### Recovery Should Address Systems Issues

The disaster exacerbated pre-existing public health problems in the community, such as widespread poor mental health and lack of affordable housing. Recovery efforts should integrate long term solutions to social drivers affecting health, e.g. social determinants of health.

### AHDs Can Help Health Departments in a Variety of Contexts

Health departments experience many interruptions of service besides natural disasters that AHD relationships could help address, such as funding shortfalls, epidemics like COVID-19, and the politicization of public health practice. In responding to crises, AHDs and students could help provide surge capacity for health departments, e.g. expanded staffing to meet increased demand for services, whether it be rolling out vaccine clinics or helping test well water.

## IMPLICATIONS

Public health education should be responsive, community-oriented, and integrated with real-world practice. The AHD model proved instrumental in enhancing local response capacity. First, students were prepared to contribute due to their embedded practicum experiences, thereby enhancing the bridge between academia and the community. Second, health departments benefited from an influx of trained volunteers with academic support. Moreover, disaster response grounded in a place-based framework respects community identity, identifies and capitalizes on local assets, and strengthens community resilience.

Academic Health Departments can be powerful engines for place-based disaster response. *Pre-established academic-practice partnerships* enabled a coordinated and community-sensitive response to Hurricane Helene, with students and faculty effectively supporting public health operations. *Investing in AHDs* enhances preparedness and builds a workforce adept in responding to complex local challenges. *Place-based models* increase trust and efficiency during emergencies.

This case study demonstrates how AHDs contribute to disaster response in a rural region, highlighting the practical benefits of integrating education with public health practice. Future research should evaluate the long-term outcomes of AHD-involved disaster responses and explore replicability in other geographic and institutional settings. Public health agencies and academic institutions should formalize and strengthen AHD partnerships, emphasizing local engagement and preparedness to address emerging public health crises.

**SUMMARY BOX**

**What is already known about this topic?**

Academic Health Departments improve public health training, foster innovation, and support evidence-based practice. Disaster preparedness is enhanced by pre-existing partnerships for coordinated response.

**What is added by this report?**

Academic Health Departments can help health departments respond quickly and effectively to natural disasters and other service disruptions by engaging students and faculty in listening to local needs, providing relevant expertise, facilitating communication with other communities who have experienced similar disasters, and providing surge staffing capacity. Disaster response grounded in a place-based framework respects community identity, identifies and capitalizes on local assets, and strengthens community resilience.

**What are the implications for future research?**

Future research should evaluate the long-term outcomes of AHD-involved disaster responses and explore replicability in other geographic and institutional settings.
